# Proyecto RiscAP: evaluación del riesgo cardiovascular en el servicio de nefrología para médicos de atención primaria

**DOI:** 10.1016/j.aprim.2025.103263

**Published:** 2025-04-08

**Authors:** José Luis Górriz, Arantxa Matalí, Sofía Sánchez, Francisco Javier Ortega Ríos

**Affiliations:** a Servicio de Nefrología, Hospital Clínico Universitario. INCLIVA. Universidad de Valencia, Valencia, España; b Medical Affairs, Boehringer Ingelheim España, Sant Cugat del Vallès, Barcelona, España; c Departamento médico de Eli Lilly and Company España, Alcobendas, Madrid, España; d Medicina de Familia, Centro de Salud Campos-Lampreana, Villarrín de Campos, Zamora, España

La enfermedad renal crónica (ERC) es, en la actualidad, un problema de salud global, cuya prevalencia en España es aproximadamente del 15%^1^. El médico de atención primaria (AP) es fundamental para detectar los factores de riesgo, de progresión y para el manejo de los estadios iniciales de la enfermedad^2^, de forma que la Sociedad Española de Nefrología (SEN) constata la necesidad de colaboración entre AP y atención especializada para prevenir y ralentizar la progresión de la ERC^2^, ^3^.

El proyecto RiscAP (Evaluación del riesgo cardiovascular en el servicio de nefrología para médicos de atención primaria) es un proyecto formativo para los médicos de AP con los objetivos de mejorar sus habilidades y su conocimiento en todos los aspectos relacionados con la ERC, y mejorar la colaboración entre las áreas de AP y de Nefrología. El proyecto contó con el aval de la SEN y del Grupo Español para el Estudio de la Nefropatía Diabética (GEENDIAB).

Se han realizado 9 ediciones de este curso, estructurado en parte teórica *(on line)* y práctica. La parte teórica incluyó aspectos relacionados con la patología, un caso clínico real y un cuestionario de evaluación. En las dos últimas ediciones se incluyeron también vídeos con puntos clave, un podcast sobre la detección precoz de la ERC y la relación bidireccional nefrólogo-AP y un cuestionario con las «30 preguntas clave que todo médico de AP debe conocer sobre la ERC». La parte práctica consistió en una formación presencial de 16 horas (distribuidas en 2-3 días) en un servicio de nefrología de un hospital cercano, en grupos de 5-6 asistentes. Durante esta formación, los alumnos pudieron asistir a las sesiones clínicas y a las consultas externas de Nefrología. Al final de cada edición se solicitó a los asistentes la cumplimentación de un cuestionario anónimo de valoración del curso.

En el total de las 9 ediciones han participado 168 hospitales y más de 1.000 médicos de AP de toda España. El 74% de los alumnos que realizaron los cursos fueron mujeres. Las comunidades autónomas en las que participaron más alumnos fueron Andalucía (15,2%), Cataluña (14,5%) y Comunidad Valenciana (14,2%).

La satisfacción media de los asistentes con el contenido del curso, valorada en una escala de 4 puntos (muy bueno: 4; bueno: 3; suficiente: 2; insuficiente: 1), constató que la utilidad práctica (compartir consulta externa con el especialista en Nefrología) y la calidad/conocimiento de los formadores fueron las actividades mejor valoradas en todas las ediciones (fig. 1) La satisfacción con el contenido del curso por parte de los formadores fue considerada como buena o muy buena por la práctica totalidad de ellos. Los resultados de los cuestionarios de las ediciones 5 a 9 mostraron que el 83,5% de los asistentes consideraban haber mejorado en todos los conocimientos, siendo el mejor valorado el manejo del paciente con ERC (51,46%), seguido de la derivación de pacientes a Nefrología (18,45%). La valoración de los docentes coincidió con la de los alumnos.

**Figura 1:**
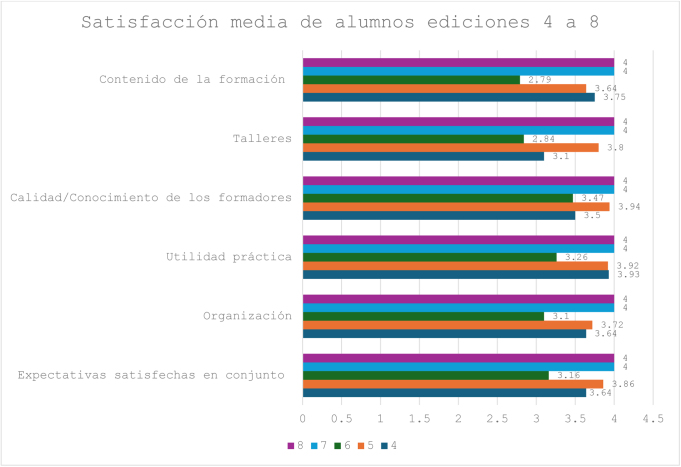
Evolución de la satisfacción media en las ediciones 4 a 8 (alumnos). (Escala de 4 puntos. Muy bueno: 4; bueno: 3; suficiente: 2; insuficiente: 1.).

El proyecto RiscAP representa un acercamiento entre la AP y la atención especializada que ha contribuido a fortalecer la comunicación entre ambas especialidades. El interés demostrado por el abordaje de la ERC denota la importancia de la detección y manejo precoz de la enfermedad. La formación de los médicos de AP puede ayudar a mejorar el cribado, el diagnóstico y el tratamiento de los pacientes con ERC, especialmente en las etapas iniciales.

En este sentido, consideramos que este proyecto cumple con el doble objetivo planteado: colaborar y mejorar las necesidades formativas que tiene el médico de AP en esta patología, y reforzar la colaboración entre AP y atención especializada.

## Financiación

El proyecto RiscAP ha sido financiado por la Alianza Boehringer Ingelheim - Lilly.

## Consideraciones éticas

El estudio se ha llevado a cabo de acuerdo con la Ley de Protección de Datos de acuerdo con la nueva Ley Orgánica 3/2018, de Protección de Datos Personales y garantía de los derechos digitales.

## Conflicto de intereses

José Luis Górriz. Honorarios por conferencias: Boehringer-Ingelheim, Lilly, Janssen, Menarini, AstraZeneca, Novartis, Novonordisk, Bayer. Advisory boards: Boehringer-Ingelheim, AstraZeneca, Bayer, Menarini.

Francisco Javier Ortega Ríos. Honorarios por ponencias, charlas, conferencias o publicaciones: Abbott, Almirall, AstraZeneca, Bayer, Biohorm, Boehringer-Ingelheim, BMS, Chiesi, Esteve, Ferrer, Glaxo-SKF, Janssen, Lilly, Menarini, MSD, Mylan, Novartis, Novex Pharma, Novo-Nordisk, Sandoz, Sanofi-Aventis, Schering-Plough, Servier, Uriach, Viatris.

Arantxa Matalí es empleada de Boehringer-Ingelheim. Sofía Sánchez es empleada de Eli Lilly.

Grupo SANED proporcionó asistencia editorial en la redacción de este manuscrito, financiado por la Alianza Boehringer Ingelheim - Lilly.

Los autores cumplen los criterios de autoría recomendados por el Comité Internacional de Editores de Revistas Médicas (ICMJE).
